# Tracing the origins of rescued chimpanzees reveals widespread chimpanzee hunting in Cameroon

**DOI:** 10.1186/1472-6785-10-2

**Published:** 2010-01-22

**Authors:** Lora Ghobrial, Felix Lankester, John A Kiyang, Akih E Akih, Simone de Vries, Roger Fotso, Elizabeth L Gadsby, Peter D Jenkins, Mary K Gonder

**Affiliations:** 1 Department of Biological Sciences, University at Albany - State University of New York, Albany, NY 12222 USA; 2 Limbe Wildlife Centre, B. P. 878, Limbe, Cameroon; 3 Wildlife Conservation Society - Cameroon Biodiversity Programme, B. P. 3055 Yaoundé, Cameroon; 4 Pandrillus, Drill Rehabilitation & Breeding Centre, H.E.P.O. Box 826, Calabar, Nigeria

## Abstract

**Background:**

While wild chimpanzees are experiencing drastic population declines, their numbers at African rescue and rehabilitation projects are growing rapidly. Chimpanzees follow complex routes to these refuges; and their geographic origins are often unclear. Identifying areas where hunting occurs can help law enforcement authorities focus scarce resources for wildlife protection planning. Efficiently focusing these resources is particularly important in Cameroon because this country is a key transportation waypoint for international wildlife crime syndicates. Furthermore, Cameroon is home to two chimpanzee subspecies, which makes ascertaining the origins of these chimpanzees important for reintroduction planning and for scientific investigations involving these chimpanzees.

**Results:**

We estimated geographic origins of 46 chimpanzees from the Limbe Wildlife Centre (LWC) in Cameroon. Using Bayesian approximation methods, we determined their origins using mtDNA sequences and microsatellite (STRP) genotypes compared to a spatial map of georeferenced chimpanzee samples from 10 locations spanning Cameroon and Nigeria. The LWC chimpanzees come from multiple regions of Cameroon or forested areas straddling the Cameroon-Nigeria border. The LWC chimpanzees were partitioned further as originating from one of three biogeographically important zones occurring in Cameroon, but we were unable to refine these origin estimates to more specific areas within these three zones.

**Conclusions:**

Our findings suggest that chimpanzee hunting is widespread across Cameroon. Live animal smuggling appears to occur locally within Cameroon, despite the existence of local wildlife cartels that operate internationally. This pattern varies from the illegal wildlife trade patterns observed in other commercially valuable species, such as elephants, where specific populations are targeted for exploitation. A broader sample of rescued chimpanzees compared against a more comprehensive grid of georeferenced samples may reveal 'hotspots' of chimpanzee hunting and live animal transport routes in Cameroon. These results illustrate also that clarifying the origins of refuge chimpanzees is an important tool for designing reintroduction programs. Finally, chimpanzees at refuges are frequently used in scientific investigations, such as studies investigating the history of zoonotic diseases. Our results provide important new information for interpreting these studies within a precise geographical framework.

## Background

Chimpanzee populations across western Africa have decreased in number by more than 75% in the last 30 years [1]; and their rate of decline is accelerating [2]. There are many reasons for this decline including the bushmeat trade [3], widespread forest clearance along with habitat alteration [1] and the spread of infectious diseases [4-6]. Capturing and smuggling live animals further exacerbates this decline [7,8]. Insufficient data and a lack of knowledge about how illegal activities directly affect chimpanzee populations impedes understanding the impact of illegal hunting on the long term survival of this species [8].

African wildlife rescue and rehabilitation projects ('refuges') have experienced a marked increase in their numbers of resident chimpanzees in the last decade [7,9]. Chimpanzees often arrive at these refuges through circuitous routes, and their geographic origins are frequently unknown [7,10]. Ascertaining the geographic origins of such chimpanzees can provide law enforcement officials with valuable insights on local patterns of wildlife hunting and smuggling [10]. For example, geographic origin estimates from large seizures of elephant ivory have provided important insights on patterns of illegal elephant harvesting and international ivory smuggling [11-13]. Some observers have suggested that trade in elephant ivory follows an 'opportunistic take' model where dealers use a decentralized plan of procuring ivory stocks to ship internationally as they become available across Africa [8]. Recent evidence suggests, however, that illegal trade in African elephant ivory may be attributed to organized crime syndicates targeting specific elephant populations for intense exploitation [12,13].

Similar to elephant exploitation, ape hunting has been proposed to follow an 'opportunistic take' model in which chimpanzees are taken by commercial hunters in the process of hunting many other species in their local areas [7,14]. However, hunters appear to be increasingly targeting apes as automatic weapons, shotguns and ammunition have become more readily available in local markets [1]. This shift towards organization and centralization in the ape trade is unsurprising as merchants can charge up to $20,000 for a live chimpanzee on the international black market [15] and roughly $100 on the local black market in Cameroon [16]. Therefore, it may be reasonable to consider the possibility that, like elephants, specific chimpanzee populations may also be targeted for intense exploitation by organized wildlife criminals. Determining the origins of rescued chimpanzees may indicate whether this pattern of targeted exploitation is shared between these two species.

The Limbe Wildlife Centre (LWC) is one of three ape rescue and rehabilitation projects that house chimpanzees in Cameroon. This refuge is home to 53 chimpanzees rescued by wildlife law enforcement officials in Cameroon as of December 2009. Until now, the geographic origins of the LWC chimpanzees have been enigmatic. Tracking the geographic origins of these chimpanzees, particularly in Cameroon, is important for two reasons. First, international wildlife crime syndicates use Cameroon as a waypoint for smuggling a variety of wildlife and wildlife-derived products (e.g., elephant ivory [12], live parrots [17] and live chimpanzees [18,19]). These illegal activities suggest that chimpanzees at the LWC may come from other countries which may complicate jurisdiction over these animals and make it difficult to enforce Cameroonian laws that prohibit hunting, capturing or selling chimpanzees and gorillas [20]. Alternatively, the LWC chimpanzees could originate in Cameroon from specific populations targeted for exploitation, as there are large local networks of hunters operating in Cameroon that target specific animal groups [21]. Therefore, illuminating where chimpanzees are procured and how they are transported could provide valuable information to Cameroonian authorities.

**Figure 1:**
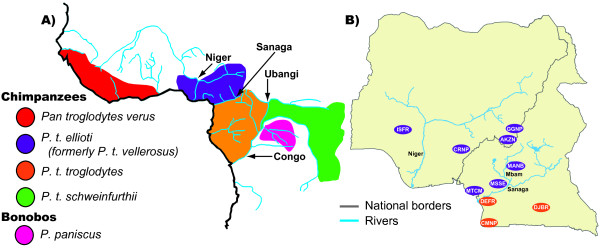
**Chimpanzee subspecies and georeferenced DNA sampling distributions**. **A) Distribution of chimpanzee subspecies**. Chimpanzees belong to a single species (*Pan troglodytes*) that is divided into four subspecies [1]. Phylogenetic analyses of mtDNA suggest that these subspecies are divided into two geographically and genetically defined groups that split about 0.5 mya: a western African group (*P. t. verus *and *P. t. ellioti *[22] [known until recently as *P. t. vellerosus *[23]) and a central/eastern African group (*P. t. troglodytes *and *P. t. schweinfurthii*) [24]. A phylogeographic break between these two groups occurs at the Sanaga River in central Cameroon, separating populations of *P. t. ellioti *north of the river from *P. t. troglodytes *south of the river. However, the Sanaga does not stop dispersal between subspecies completely because some gene flow between them occurs near the confluence of the Sanaga and its main tributary, the Mbam River [24,28]. **B) Map of Cameroon and Nigeria showing collection sites of georeferenced chimpanzee DNA samples**. Sampling sites shown on the map are: Ise Forest Reserve (ISFR), Cross River National Park (CRNP), Akoh Zanto (AKZN), Gashaka Gumti National Park (GGNP), Mount Cameroon (MTCM), Mosse (MSSE), Manb'ra (MANB), Douala-Edea Forest Reserve (DEFR), Campo-Ma'an National Park (CMNP) and Dja Biosphere Reserve (DJBR).

Second, Cameroon is home to two chimpanzee subspecies (Figure 1a), *Pan troglodytes ellioti *[22] (known until recently as *P. t. vellerosus *[23]) and *P. t. troglodytes *[1,24,25], although the taxonomy of chimpanzee subspecies is debated [26,27]. The ranges of these two subspecies converge at the Sanaga River in central Cameroon, which acts as a barrier to their dispersal; despite this, some limited gene flow between the two subspecies occurs around the confluence of the Sanaga River and its main tributary, the Mbam River [28,29]. Reintroduction programs are being developed for these chimpanzees and must take into account the genetic histories of their chimpanzees in order to be most effective [30]. The rich and complex biogeographic history of chimpanzees in Cameroon make evaluating the actual location of these reintroductions important towards maintaining evolutionary significant units of this species [7,9,30]. In particular, *P. t. ellioti *has a very restricted range, occurring only in Nigeria and western Cameroon [1]; and 6,000-10,000 are believed to persist in the wild [31].

We addressed three questions in this study. First, are the LWC chimpanzees from Cameroon? Second, if so, do their origins correspond to biogeographic boundaries for this species in Cameroon? Finally, do these data suggest that hunting is widespread; and/or do hunting 'hotspots' exist in Cameroon where focused law enforcement is needed? We addressed these questions by estimating the geographic origins of 46 chimpanzees housed at the LWC using Bayesian approximation approaches [11]. We compared microsatellite (STRP) loci genotype profiles and mtDNA sequence data against a spatial map of allele frequencies constructed from orthologus genotypes from georeferenced chimpanzee DNA samples from ten locations spanning Cameroon and Nigeria (Figure 1b). The LWC chimpanzees were estimated to be from one of three biogeographically important zones within Cameroon (or adjacent parts of Nigeria). The majority of them were estimated to originate within the range of *P. t. ellioti*, but several were also estimated to originate within the range of *P. t. troglodytes*. Their estimated origins are dispersed across the country, suggesting that hunting is widespread across Cameroon. Although our current sample size of rescued chimpanzees is relatively small, these data suggest that, unlike patterns of organized elephant hunting, chimpanzee hunting in Cameroon may follow an 'opportunistic take' model. More data from a broader sample of rescued chimpanzees should be compared against a more inclusive grid of georeferenced chimpanzee samples before definitive conclusions about chimpanzee exploitation in Cameroon may be drawn.

## Results and Discussion

### STRP genotype profile dataset

A total of 185 chimpanzee DNA samples were typed at ten autosomal STRP loci for this study. Forty-six of these samples were from LWC chimpanzees, whereas 139 were from georeferenced chimpanzee DNA samples from Cameroon and Nigeria. We considered an STRP profile suitable for analysis if it included six or more loci. A total of 88% of the 185 samples had suitable STRP profiles and were included in all assignment tests, but the vast majority of these samples had reliable allele scores for at least eight loci. In total, all 46 LWC chimpanzee STRP profiles were used in the assignment tests, whereas 86 STRP profiles from georeferenced chimpanzee DNA samples from ten locations across the study area (Figure 1b) were used in all our assignment tests. STRP allele sizes for samples included in this study are listed in Additional File 1.

### mtDNA haplotype analysis

**Figure 2:**
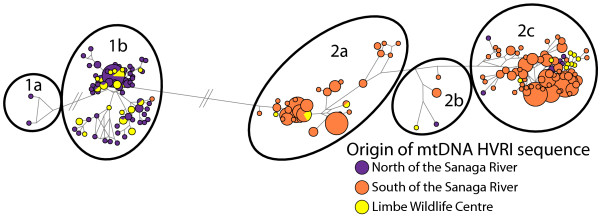
**Median joining network of mtDNA HVRI sequences**. This network is composed of 464 georeferenced chimpanzee samples spanning Cameroon and Nigeria that were reported in previous studies [22,23,26-28] and LWC chimpanzee samples (n = 46). Haplotypes were color coded denoting their region of origin. Samples shown in purple were collected in Nigeria and western Cameroon west and north of the Sanaga River. Samples shown in orange were collected in southern Cameroon south of the Sanaga River.

Sequences of the first hypervariable region (HVRI) of mtDNA were newly generated for each of the 46 LWC chimpanzees and aligned against a georeferenced dataset composed of 464 HVRI sequences from 28 locations across Nigeria and Cameroon from previous studies [23,24,32-34] that are publically available on DDBJ/EMBL/GenBank International Nucleotide Sequence Database. The median joining network shown in Figure 2 was partitioned into two major haplotypes. Haplotype 1 was composed of georeferenced chimpanzee samples within the range of *P. t. ellioti *from Nigeria and western Cameroon north of the Sanaga River (including those from the transition zone in central Cameroon), with a single exception. Haplotype 1 was subdivided into two subsets. Haplotype 1a was composed of georeferenced samples from western Nigeria, whereas Haplotype 1b was composed of georeferenced chimpanzee samples from eastern Nigeria and western Cameroon. Thirty-two of the LWC chimpanzees clustered into Haplotype 1b. Haplotype 2 was composed mostly of georeferenced samples that were collected in southern Cameroon south of the Sanaga River within the range of *P. t. troglodytes*. Haplotype 2 was further subdivided into three subsets: Haplotypes 2a, 2b and 2c. These haplotypes were composed mostly of samples collected at the same, or nearby, sampling locations in southern Cameroon. Fourteen of the LWC chimpanzees clustered within Haplotype 2, of which four, one and nine clustered into Haplotypes 2a, 2b and 2c, respectively. These mtDNA haplotype designations (1a, 1b, 2a, 2b and 2c) were encoded as an eleventh locus in each assignment test for all the LWC chimpanzees and the 86 georeferenced chimpanzee STRP profiles.

### SAM assignment tests

We performed assignment tests for each individual using smoothed and continuous assignment methods [11], implemented in the SCAT software program http://stephenslab.uchicago.edu/software.html. SCAT uses allele frequencies from georeferenced samples combined with spatial smoothing methods to generate a geographic map of allele frequency variation. The smoothed assignment method (SAM) combines smoothed reference maps of allele frequency variation and an MCMC algorithm to generate a posterior distribution of the probability that samples of unknown origin share ancestry with samples of known origin from the allele frequency variation map of georeferenced samples [11].

We first explored the reliability of the SAM at accurately estimating the origins of our georeferenced samples by a leave-one-out cross-validation procedure in which each sample in turn was treated as the sample whose location was unknown, whereas the other samples were assumed to have known location. Results of the cross-validation tests are given in Table 1. We assessed the reliability of the SAM in two ways. First, we assessed how accurately the SAM assigned georeferenced samples to their *region *of origin (i.e., from a location that is either north or south of the Sanaga River). The SAM accurately placed individuals as originating north versus south of the Sanaga River in 89% of assignments. Half of the samples that were not placed back to their actual region of origin came from MANB, a location lying in the transition zone where limited gene flow appears to occur between the two subspecies of chimpanzees in central Cameroon. Second, we assessed how reliably the SAM assigned georeferenced samples to their *location *of origin. The SAM accurately estimated sampling location origin in 58% of assignments. Some locations [e.g., Mount Cameroon (MTCM), Ise Forest Reserve (ISFR), and to a lesser extent, areas in and near the Cameroon Highlands (CRNP, AKZN and GGNP)] had a much higher proportion of samples correctly estimated back to their true locations, suggesting that chimpanzees at these locations were genetically somewhat distinct from those at other locations we sampled. Many of the samples with incorrect SAM assignments had estimated origins at locations very close to their actual sampling location. Nearly all samples with incorrect SAM assignments were estimated to have come from locations within the same geographic zone (i.e., north of the Sanaga, the transition zone or south of the Sanaga).

**Table 1:** Geographic origins of samples inferred using the SAM

Georeferenced SAM assignments											% Accuracy of georeferenced SAM assignments		LWC SAM assignments
	ISFR	GGNP	AKZN	CRNP	MSSE	MTCM	MANB	DEFR	CMNP	DJBR	Location^1,2^	Region^3,4^	
ISFR	3	0	0	0	1	0	0	0	0	0	75	100	0
GGNP	0	6	1	0	1	2	0	0	0	0	60	100	9
AKZN	0	1	4	2	3	1	0	0	0	0	36	100	1
CRNP	0	0	1	5	0	1	0	0	0	1	63	88	1
MSSE	0	1	1	0	5	1	0	0	0	0	63	100	6
MTCM	0	0	1	0	0	10	0	0	0	0	91	100	15
MANB	0	0	0	0	0	1	4	4	1	0	40	50	3
DEFR	0	0	0	1	0	0	2	4	0	1	50	63	0
CMNP	0	0	0	0	0	0	0	0	5	3	63	100	4
DJBR	0	1	0	0	0	0	0	3	2	4	40	90	7

^1^Percentage of georeferenced sample assignments using the SAM that were correctly assigned to their actual sampling location of origin.

^2^Average accuracy, 58%.

^3^Percentage of georeferenced sample assignments using the SAM that were correctly assigned back to their regional division but not necessarily their correct sampling location of origin. Two regions were considered: 1) sampling locations that lie north and west of the Sanaga River in Nigeria and western Cameroon [ISFR, GGNP, AKZN, CRNP, MSSE, MTCM and MANB] and 2) sampling locations that lie south of the Sanaga River in southern Cameroon [DEFR, CMNP and DJBR]. Samples from the transition zone (MANB) in central Cameroon were grouped with samples north of the Sanaga to obtain these estimates.

^4^Average accuracy, 89%.

Given the results of the SAM reliability tests, we concluded that the SAM should be very accurate for estimating whether the LWC samples originated from either the region north and west of the Sanaga River or from the region south of the Sanaga River. Furthermore, we expected the SAM to produce less reliable results when estimating origins within those two regions given the mixed performance of the SAM at accurately placing the georeferenced samples to their correct locations within these two regions. Finally, these results suggest that there is substantial population structure separating populations north and south of the Sanaga River, but less population structure between populations within these regions, as expected based on previous studies [29].

We used the SAM to estimate an origin of each LWC chimpanzee. These point estimates were determined by the highest log-likelihood ratios of the posterior distribution that each sample originated from a particular location across five independent runs for a total of 10,000 iterations for each sample. Summaries of these assignments are given in the last column of Table 1, whereas details regarding SAM assignments for each LWC chimpanzee are given in Table 2. Forty-two of the LWC samples were consistently assigned the same estimated origin across independent runs. Only four of the LWC chimpanzees had SAM location estimates that varied across runs; however, the discrepancies between the SAM estimated origins of those four samples were from sampling locations that lie relatively close to each other and in the same regional partition. Table 2 shows the majority consensus for these SAM origins across independent runs. The SAM assignment estimates revealed that 35 of the LWC chimpanzees originated from north of the Sanaga River, whereas 11 originated from south of the Sanaga River. Interestingly, a high proportion of LWC chimpanzees were estimated to have come from Mount Cameroon (MTCM) and Gashaka Gumti National Park (GGNP). MTCM and GGNP are two locations where the SAM reliability tests accurately estimated the sampling location origins of the georeferenced samples most frequently.

**Table 2:** Summary of origin estimates for the LWC chimpanzees

LWC ID	mtDNA Haplotype^1^	SAM Location^2^	SAM Region^3^	CAM Coordinates^4^		CAM Region^5^	Postulated Subspecies^6^
001	1b	MTCM	North	5.69426	10.98525	North	*P. t. ellioti*
002	1b	MTCM	North	4.79855	9.2901	North	*P. t. ellioti*
003	1b	MTCM	North	5.88904	10.14165	North	*P. t. ellioti*
004	1b	GGNP	North	6.06406	10.804	North	*P. t. ellioti*
005	1b	MSSE	North	6.01814	9.33696	North	*P. t. ellioti*
006	1b	MTCM	North	6.16266	9.770035	North	*P. t. ellioti*
007	1b	GGNP	North	6.4109	10.4424	North	*P. t. ellioti*
008	1b	MTCM	North	5.508405	10.144	North	*P. t. ellioti*
009	1b	MSSE	North	6.02645	10.4546	North	*P. t. ellioti*
010	1b	GGNP	North	6.686045	11.5976	North	*P. t. ellioti*
011	1b	MTCM	North	4.999935	9.59359	North	*P. t. ellioti*
012	1b	GGNP	North	6.387525	10.6643	North	*P. t. ellioti*
014	1b	GGNP	North	6.171715	11.5504	North	*P. t. ellioti*
017	1b	GGNP	North	6.20374	11.4116	North	*P. t. ellioti*
018	1b	MTCM	North	5.71996	9.154565	North	*P. t. ellioti*
019	1b	GGNP	North	6.819025	10.63615	North	*P. t. ellioti*
021	1b	GGNP	North	6.78872	11.53365	North	*P. t. ellioti*
023	1b	MSSE	North	5.784825	10.3854	North	*P. t. ellioti*
024	1b	MTCM	North	4.15989	9.14837	North	*P. t. ellioti*
026	1b	MSSE	North	5.74762	10.60665	North	*P. t. ellioti*
028	1b	MTCM	North	4.3105	9.169405	North	*P. t. ellioti*
030	1b	MTCM	North	5.691485	10.45	North	*P. t. ellioti*
033	1b	MTCM	North	5.88719	9.870615	North	*P. t. ellioti*
034	1b	AKZN	North	6.09503	11.2155	North	*P. t. ellioti*
036	1b	MTCM	North	5.709555	10.8607	North	*P. t. ellioti*
038	1b	MSSE	North	5.240895	10.1503	North	*P. t. ellioti*
041	1b	MTCM	North	5.97631	9.97306	North	*P. t. ellioti*
043	1b	GGNP	North	6.637165	10.9659	North	*P. t. ellioti*
044	1b	MTCM	North	6.04163	9.87016	North	*P. t. ellioti*
045	1b	MTCM	North	5.55618	9.42	North	*P. t. ellioti*
046	1b	MTCM	North	5.767895	10.37835	North	*P. t. ellioti*
016	1b	MANB	North	5.616985	11.6057	Transition	*P. t. ellioti*
027	2a	DJBR	South	3.47309	12.80025	South	*P. t. troglodytes*
031	2a	DJBR	South	3.933035	13.092	South	*P. t. troglodytes*
032	2a	CMNP	South	3.65541	11.2014	South	*P. t. troglodytes*
040	2a	MANB	North	5.366435	11.657	Transition	*P. t. troglodytes*
015	2b	CRNP	North	5.947675	11.5971	Transition	*P. t. troglodytes*
025	2c	DJBR	South	5.077715	11.7409	Transition	*P. t. troglodytes*
013	2c	CMNP	South	3.578515	12.5198	South	*P. t. troglodytes*
020	2c	CMNP	South	3.364205	12.05345	South	*P. t. troglodytes*
022	2c	DJBR	South	3.3743	12.344	South	*P. t. troglodytes*
029	2c	DJBR	South	4.137805	12.88305	South	*P. t. troglodytes*
035	2c	DJBR	South	4.54397	12.2347	South	*P. t. troglodytes*
037	2c	CMNP	South	4.33148	11.3812	South	*P. t. troglodytes*
042	2c	DJBR	South	3.38591	12.71325	South	*P. t. troglodytes*
039	2c	MSSE	North	4.7324	10.4764	North	Uncertain

^1^mtDNA haplotype inferred from the median-joining network in Figure 2.

^2^Estimated sampling location origin of sample using the SAM.

^3^Estimated regional location using the SAM. Here, samples were grouped by region into locations that are either north and west of the Sanaga (locations shaded purple in Figure 1b) or south of the Sanaga River (locations shaded orange in Figure 1b).

^4^Point estimates of origins using the CAM. Point estimates are listed in decimal coordinates.

^5^Point coordinate estimates were grouped by region: 1) north and west of the Sanaga (North), 2) south of the Sanaga River in southern Cameroon (South) or 3) the transition zone near the confluence of the Sanaga and Mbam Rivers (Transition).

^6^Our estimate of subspecies designation based on CAM estimated origins and mtDNA haplotype.

### CAM assignment tests

**Figure 3:**
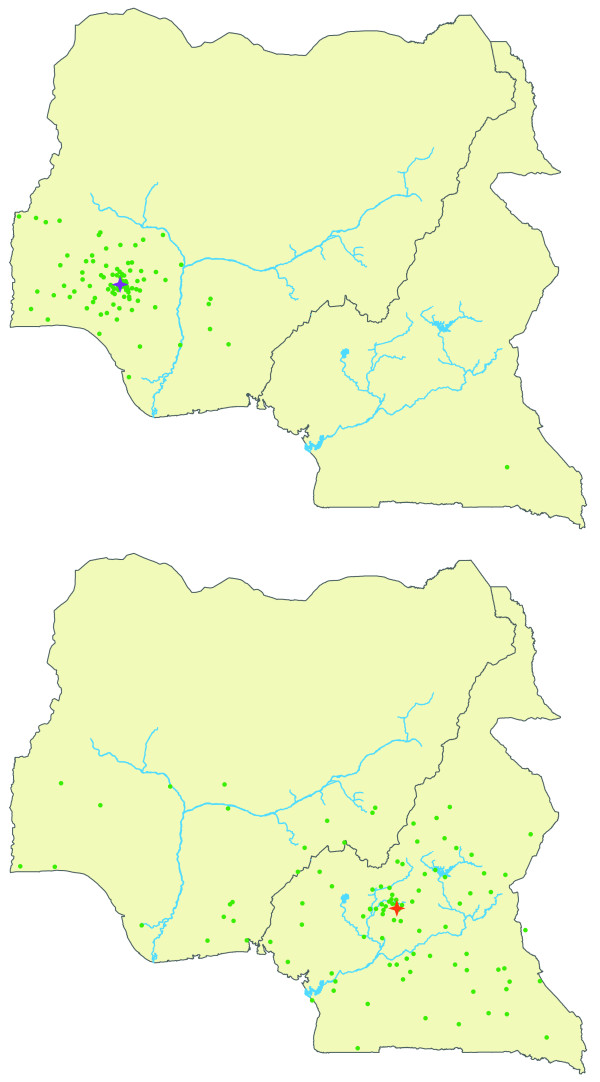
**Representation of confidence in CAM assignments for georeferenced chimpanzee samples**. The green circles represent 100 point estimates drawn randomly from the set of all possible locations weighted according to their probability. Stars represent the median point coordinate estimate for each georeferenced sample. The concentration of these 100 circles in any given area is a guide to the probability that the sample arose from that area, where tighter concentrations of circles indicate higher confidence in the median point estimate. The top panel shows a georeferenced sample (ISFR001) with estimated CAM origins near its actual location of origin, along with a high concentration of circles near its location origin. The bottom panel shows a georeferenced sample with estimated CAM origins also near its actual sampling location (MANB014), but with more dispersed coordinate point estimates.

We also performed assignments using a continuous assignment method (CAM). The CAM is a considerable improvement over traditional assignment tests in that estimated origins are independent of sampling locations included in the study. In particular, the CAM returns point estimates of geographical coordinates for each unknown sample that can be from anywhere within a specified geographic boundary [11]. In this case, we allowed the CAM to estimate an origin anywhere within suitable chimpanzee habitat across the study area. We assessed the reliability of the CAM by calculating the median values of 10,000 coordinate point estimates for each georeferenced sample from five independent CAM runs with a leave-one-out cross-validation check. Then we estimated the accuracy of these estimates in two ways. First, we plotted 100 coordinates that were drawn randomly from the set of all possible locations across the full 10,000 CAM estimates for each georeferenced sample. Tighter clustering of points indicates higher confidence in the median point coordinate estimates, whereas increased point dispersion indicates lower confidence in a given georeferenced chimpanzee sample's estimated CAM origin. We generally observed more geographically concentrated clusters of point coordinate estimates in chimpanzees originating from north of the Sanaga River, as opposed to those from south of the Sanaga River or from the transition zone. Examples of the patterns observed in these plots are shown in Figure 3.

Second, we calculated the straight line distances between the estimated CAM origin of each georeferenced sample and the sample's *actual *location coordinates. This CAM reliability test showed that 30%, 50% and 80% of the georeferenced samples could be accurately placed within 93 km, 157 km and 254 km of their *actual *sampling location of origin, respectively. Furthermore, there are three zones in Cameroon that correspond to different 'pockets' of diversity observed from patterns in the georeferenced samples discussed in previous studies [24]: 1) north and west of the Sanaga River, 2) south of the Sanaga in southern Cameroon and 3) a transition zone around the confluence of the Sanaga and Mbam Rivers that is not well understood. We partitioned all straight line distance estimates into these three biogeographically important zones. Based on these criteria, 85% of the georeferenced samples had straight line distances between their actual origin and their estimated origin that occurred completely within the zone where the samples originated. The vast majority of georeferenced samples placed in the wrong zone had estimated origins in the transition zone. Based on these observations, we concluded that the CAM should be very accurate for estimating whether the LWC chimpanzees originated north of the Sanaga River, south of the Sanaga River and, possibly, from the transition zone. However, we expected that the CAM would produce less reliable point estimates at a more fine-grained geographic scale.

**Figure 4:**
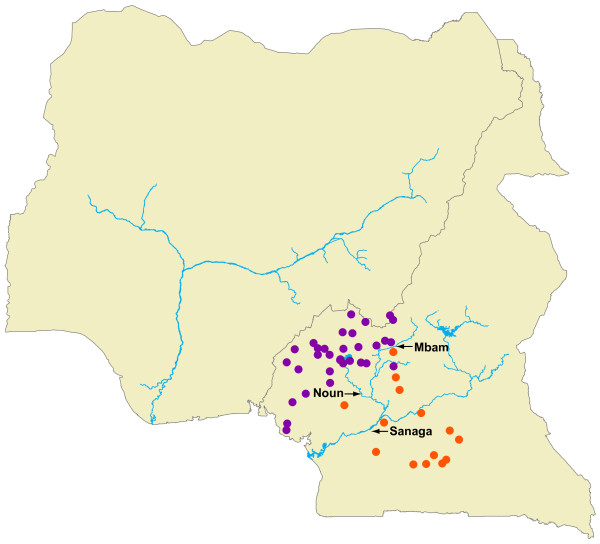
**Estimated CAM origins of 46 LWC chimpanzees**. Samples were color coded to denote their mtDNA haplotype membership shown in Figure 2. Samples shown in purple clustered with mtDNA Haplotype 1, whereas those shown orange clustered with mtDNA Haplotype 2.

A plot of median coordinate point estimates for each LWC chimpanzee is shown in Figure 4 and are also listed in Table 2 in decimal degrees. All of the LWC chimpanzees have estimated origins in Cameroon, with the exception of one that may originate from near the Mambilla Plateau that straddles the Cameroon-Nigeria border. We further partitioned the LWC chimpanzees' estimated CAM origins into the three biogeographically important zones across the region. Samples shown in Figure 4 were also color-coded according to their mtDNA haplotype membership as determined by the median-joining network analysis (Figure 2). Samples shown in purple clustered with georeferenced samples from north and west of the Sanaga River (Haplotype 1b), whereas samples colored orange clustered with samples mostly from southern Cameroon south of the Sanaga River (Haplotypes 2a, 2b and 2c). Samples with estimated CAM origins in the transition zone in central Cameroon consisted of both mtDNA haplotypes (i.e., purple and orange circles). These findings support previous georeferenced population genetic data suggesting that some introgression occurs between *P. t. ellioti *and *P. t. troglodytes *around the confluence of the Mbam and Sanaga Rivers [24,28]. The mtDNA haplotype analysis was consistent with CAM assignments in 87% of the tests in placing the LWC chimpanzees as occurring either north or south of the Sanaga.

**Figure 5:**
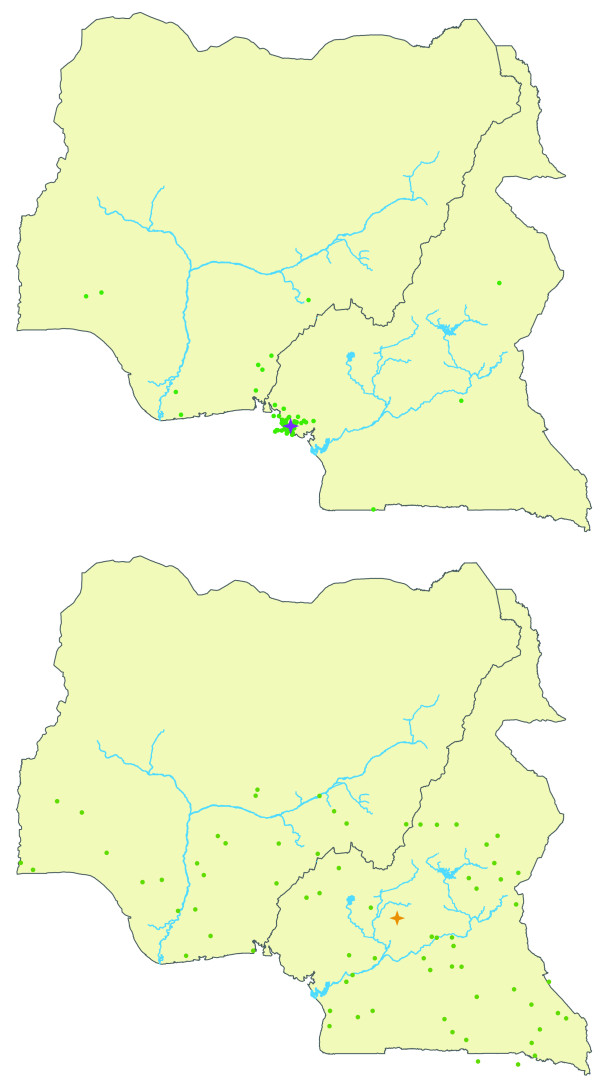
**Representation of confidence in CAM assignments for LWC chimpanzee samples**. The green circles represent 100 point estimates drawn randomly from the set of all possible locations weighted according to their probability. Stars represent the median point coordinate estimate for each sample, color coded according to each sample's mtDNA haplotype from Figure 2. The concentration of these 100 circles in any given area is a guide to the probability that the sample arose from that area, where tighter concentrations of circles indicate higher confidence in the median point estimate. The top panel shows LWC026 with a tight concentration of circles near its median point estimate. The bottom panel shows LWC040 with more dispersed coordinate point estimates.

Ten LWC chimpanzees that had mtDNA Haplotype 2 were also assigned origins south of the Sanaga. Thirty-one LWC chimpanzees that had mtDNA Haplotype 1b had also CAM estimated origins north of the Sanaga. One LWC chimpanzee (LWC039) had an estimated CAM origin north of the Sanaga but also belonged to mtDNA Haplotype 2c. LWC039 was rescued near Bertoua, Cameroon (N 4.5753 E 13.6847), which lies near where the Lom and Pangar Rivers merge to form the Sanaga River in eastern Cameroon. Analyses of an additional 27 STRP loci of LWC039 compared to chimpanzees representing each subspecies indicated that this chimpanzee shares significant ancestry with both *P. t. ellioti *and *P. t. troglodytes *(Gonder, Ghobrial and Locatelli, unpublished results). Based on this information, we suspect that adding more STRP loci to the CAM tests will eventually place LWC039 inside the transition zone. The remaining four LWC chimpanzees have CAM estimated origins in the transition zone. We also examined how much confidence we could place in each estimated origin by plotting 100 coordinates that were drawn randomly from the set of all possible locations across the full 10,000 CAM estimates for each LWC chimpanzee, with the degree of spread indicating how much confidence to give to any one CAM estimated origin. Examples of these plots are shown in Figure 5. Generally, we observed tighter clustering of points in samples from western Cameroon than in those estimated to be from the transition zone or southern Cameroon. Moreover, we did not observe any greater dispersion of points in the LWC samples compared to our confidence plots for the georeferenced dataset.

These observations suggest that the LWC chimpanzees come from many areas across Cameroon, but the majority appear to have come from within the range of *P. t. ellioti*. We also calculated straight line distances between each estimated origin and the nearest protected area. These calculations revealed that 24 of the LWC chimpanzees had estimated origins either inside or < 75 km from a protected area. These findings suggest that chimpanzee hunting is relatively widespread across Cameroon and occurs in both protected and unprotected areas. In addition, live animal transport appears to occur locally within Cameroon.

### CAM estimated origins in southern Cameroon

One limitation of the CAM is that this method requires that a boundary be specified of allowable locations across a continuous region. In other words, all samples of unknown origin are assumed to have originated within the specified boundary, which can be problematic if reference samples are unavailable for a portion of a species' range. For our first CAM assignments, we specified a boundary that included Nigeria and Cameroon, the range of our georeferenced samples. We had more confidence in the results for the LWC chimpanzees we estimated to belong to *P. t. ellioti *for three reasons. First, we included reference samples that covered the complete range of *P. t. ellioti*. Second, all LWC chimpanzees that belonged to mtDNA Haplotype 1b, a haplotype used to identify *P. t. ellioti *in other studies [32-35], also had CAM origins from north of the Sanaga in the range of *P. t. ellioti *or the transition zone. Third, the LWC and georeferenced chimpanzee samples estimated to originate north of the Sanaga River were more tightly clustered in the coordinate plots compared to those from southern Cameroon, suggesting higher confidence in those assignments. However, our reference samples for *P. t. troglodytes *come only from southern Cameroon, which is only a small portion of the range of that subspecies. Due to this small reference sample, we explored how likely it was that the LWC chimpanzees with estimated origins from southern Cameroon might have come from somewhere outside southern Cameroon but within the larger range of *P. t. troglodytes*.

We created a second boundary file that included the complete range of *P. t. troglodytes *across central Africa and a data file containing only georeferenced data from southern Cameroon. In this new CAM test, we used georeferenced samples from western Nigeria as negative controls for the new boundary file, since we knew their origin to be outside the range of *P. t. troglodytes*. We also challenged this CAM test to estimate the origins of LWC chimpanzees originally placed in southern Cameroon by the Cameroon-Nigeria bounded CAM tests; except in this test these LWC samples were allowed to originate anywhere within the range of *P. t. troglodytes*. We plotted 100 coordinates that were drawn randomly from the set of all possible locations across 2,000 CAM coordinate point estimates for each sample included in this test. We expected the point estimate coordinate plots for the georeferenced samples from western Nigeria to be more dispersed across the area than the plots for samples that were more likely to have originated within the specified boundary (i.e., those with estimated origins in southern Cameroon).

Point estimate coordinate plots for the georeferenced samples from western Nigeria were very dispersed across the entire boundary specified for the CAM. The plots for the LWC chimpanzees generally showed more clustering in southern Cameroon as opposed to other areas in the range of *P. t. troglodytes*. T-test comparisons revealed that the LWC samples had significantly more coordinate point estimates in southern Cameroon (p < 0.01) than in other parts of the range of *P. t. troglodytes *compared to the control samples from western Nigeria. These observations suggest that it is more likely that the LWC chimpanzees estimated to be from southern Cameroon by the original CAM tests are indeed more likely to be from southern Cameroon than other regions within the range of *P. t. troglodytes*. However, some degree of uncertainty regarding the origins of these ten LWC chimpanzees is warranted.

## Conclusions

In this study, we demonstrate that the LWC chimpanzees originate in Cameroon or contiguous forests along the Cameroon-Nigeria border. Second, we have considerable power using both SAM and CAM tests to assign each LWC chimpanzee to one of three biogeographically important zones in Cameroon: north of the Sanaga River, south of the Sanaga River or from the transition zone in central Cameroon. The SAM and CAM appeared to have less power for estimating origins on a finer geographic scale within these biogeographically important zones. Intriguingly, these data provide additional support for the hypothesis that introgression between *P. t. ellioti *and *P. t. troglodytes *occurs around the confluence of the Mbam and Sanaga Rivers in central Cameroon [24,28]. We are conducting extensive population genetic studies of chimpanzees from this region to verify these observations in a larger, fully georeferenced data set.

The CAM estimated origins indicate that the majority of the LWC chimpanzees appear to have come from western Cameroon within the range of *P. t. ellioti *(n = 32) or from the transition zone (n = 4), which is unsurprising as the wildlife protection authorities based at the LWC focus their rescue and seizure efforts in that area of Cameroon. Ten LWC chimpanzees are likely to come from southern Cameroon within the range of *P. t. troglodytes*. Although we have limited power with this sample to make very firm conclusions, these data are suggestive of trends in patterns of chimpanzee hunting and live animal smuggling in Cameroon. Chimpanzee hunting and live animal transport largely appears to occur locally within Cameroon, despite the existence of well organized wildlife crime cartels in the country that operate internationally [8,13]. That is, we did not find evidence to suggest that chimpanzees are being transported over large distances, involving movement over national borders prior to their residence at refuges (with the exception of potential border-crossing between Cameroon and Nigeria). Both assignment test methods reveal also that the LWC chimpanzees come from many areas across Cameroon and near the Cameroon-Nigeria border. These observations indicate that chimpanzee hunting is widespread in Cameroon. In addition, the CAM assignments suggest that LWC chimpanzees come from both protected and unprotected areas of Cameroon suggesting that local legal protection across the country needs to be reinforced. Given that 10 chimpanzees may be killed for each chimpanzee that survives in a sanctuary [1], this evidence of widespread hunting underestimates the full extent of chimpanzee exploitation in Cameroon.

These observations make it difficult to pinpoint chimpanzee hunting 'hotspots', if they exist, given our current sample. It is possible that chimpanzee hunting 'hotspots' may not exist in Cameroon because these animals are taken when it is advantageous for the hunter as in the 'opportunistic take' model. However, the fact that we did not observe chimpanzee hunting 'hotspots' in Cameroon may be attributable to two factors. First, the LWC sample was relatively small making it difficult to pinpoint potential hunting 'hotspots' if they do exist. Plans are underway to include chimpanzees from other refuges in similar studies in the near future to search for more specific trends in chimpanzee hunting in Cameroon from a larger sample. Second, a more comprehensive grid of georeferenced chimpanzee samples that includes genotype profiles at more STRP loci may make it possible to increase the precision of origin assignments using the SCAT approach.

Our findings offer promising insights that may augment assessing the location(s) of reintroducing the LWC chimpanzees back into their natural habitats. IUCN guidelines suggest that whenever possible, apes should be reintroduced within their historical range to the lowest 'unit of conservation action' [30]. Our results indicate that Cameroon is the appropriate location for reintroducing these chimpanzees back into the wild as they all appear to be from the area. Furthermore, their estimated CAM origins partition closely with the ranges of the two chimpanzee subspecies occupying Cameroon [24], which suggests that it may be possible to reintroduce these chimpanzees back into their historical ranges.

Finally, chimpanzees at refuges are frequently used in scientific investigations, such as studies that focus on illuminating the history of zoonotic diseases like SIV*cpz *[35,36] and malaria [37]. These studies are often hampered by a lack of knowledge about the geographic origins of these chimpanzees [38]. Several studies have shown that chimpanzee population history can be very important for understanding the distribution of disease [32-34], but these studies have encountered considerable obstacles owing to the difficulties of working with fecal samples from reclusive, highly endangered chimpanzee populations [32]. Refuge chimpanzees are a unique reservoir for understanding both the history of this species and of zoonotic diseases because they reside in an environment where it is possible to obtain high-quality samples for extensive analysis. Our results provide a foundation for interpreting the findings of these studies within a precise geographical framework. In conclusion, these data reveal that illuminating the uncertain origins of refuge chimpanzees using the SCAT approach is a powerful tool that can provide valuable information to local wildlife law enforcement personnel for ascertaining patterns and trends in chimpanzee hunting, for planning reintroduction programs and for informing scientific investigation involving these chimpanzees.

## Methods

### DNA sample collection and isolation

Veterinarians at the LWC collected whole blood from 46 chimpanzees, during routine health checkups. Georeferenced chimpanzee hair samples were selected from a collection of samples recovered from abandoned sleeping nests from ten locations throughout Cameroon and Nigeria reported in previous studies [24,29]. All samples were transported from Cameroon to the United States in full compliance with CITES and CDC export and import regulations. This research was carried out with IACUC approval from the University at Albany, State University of New York. DNA was isolated from whole blood of 46 chimpanzees at the LWC veterinary clinic, using well-established salting out procedures [39]. These samples yielded a range of 31-1098 ng/μl of DNA. DNA from the hair samples was extracted using a chelating resin protocol [24] followed by filtration using Microcon 100 columns (Millipore - Billerica, MA) to concentrate DNA extracts.

### STRP genotyping and allele size verification

Ten STRP loci were used to produce genotype profiles from both the georeferenced dataset from previous studies [24] and the 46 LWC chimpanzees. Table 3 lists the markers chosen labeled with the G5 fluorescent dye set (Applied Biosystems, Foster City, CA) necessary for multiplexing the ten loci into two multiplex PCR reactions [40,41]. PCR reactions were performed using the Qiagen Multiplex PCR Kit (Qiagen, Valencia, CA) in Eppendorf Mastercyclers (Eppendorf, Westbury, NY). PCR reactions involving blood DNA extracts were carried out using the manufacturer's protocol and 1 ng of DNA for each reaction. PCR reactions involving hair DNA extracts were carried out using 0.5 - 1 ng DNA, along with Q-Solution (provided in the kit) and a 5-10 additional 3-step thermocycles [40,41]. PCR conditions for the georeferenced hair samples were: 95°C for 15 min, 40 cycles of 94°C for 30 s, 60°C for 90 s, 72°C for 1 min, and a final extension of 60°C for 30 min. Although many of the hair samples had been typed previously [29], each of the georeferenced samples were retyped for this study to avoid differences in base pair sizes due to apparatus and protocol discrepancies [42]. All PCR reactions included negative control samples for quality assurance.

**Table 3:** STRP markers included in this study

Marker	Repeat Unit	Size Range (bp)	Primer Sequences (5'-3')
Mfd3	AC	116-158	F - VIC - GGT CTG GAA GTA CTG AGA AAA
			R - GAT TCA CTG CTG TGG ACC CA
Mfd23	AC	73-123	F - VIC - CCA GAC ATG GCA GTC TCT A
			R - AGT CCT CTG TGC ACT TTG T
HumPla2a	AAT	70-104	F - 6FAM - GGT TGT AAG CTC CAT GAG GTT AGA
			R - GTC CTA GGA GCT AGA GAT ACA GC
D4S1652	ATCT	105-165	F - 6FAM - AAT CCC TGG GTA CAT TAT ATT TG
			R - GGA GGT AAA GAA TAA AGA ATG TCT G
D7S1809	AGGA	192-256	F - 6FAM - AGG CAA GAG CAG TAG CAA GA
			R - TCC ACT TTA AAT CAG CAG CC
D9S303	GATA	137-193	F - NED - CAA CAA AGC AAG ATC CCT TC
	xCAGA		R - GCC AAG AGT TTC CAA GTA CCT A
D11S1984	CAAA	99-207	F - PET - GGG TGA CAG AGC AAA ATT CT
			R - ACA CCT GGA TCT TGG ACT CA
D13S317	TATCx	152-252	F - VIC - ACA GAA GTC TGG GAT GTG GA
	ATCT		R - GCC CAA AAA GAC AGA CAG AA
D16S539	ACAGx	134-165	F - 6FAM - GAT CCC AAG CTC TTC CTC TT
	GATA		R - ACG TTT GTG TGT GCA TCT GT
D20S470	TTCCxCCTTx	193-321	F - PET - CCT TGG GGG ATA TAG CCT AA
	CCTTxTC		R - CAT GGT ATC ACT CTG TCA CTC A

Each multiplex PCR product was analyzed on an ABI 3130 capillary array genetic analyzer (Applied Biosystems, Foster City, CA). Fragment sizes were determined against Genescan 600 Liz size standard (Applied Biosystems, Foster City, CA). Allele sizes were determined using the Genemapper ID version 2.7 software (Applied Biosystems, Foster City, CA). Alleles were scored between two and four times to avoid problems associated with allelic dropout which frequently occurs when genotyping low-yield DNA samples [43]. Samples that did not include six or more loci after multiple attempts at PCR fragment amplification were excluded from this study.

### mtDNA HVRI resequencing

The HVRI of mtDNA was resequenced in each of the LWC chimpanzees from a 10.6 kb PCR fragment to reduce problems associated with NUMTs [44,45] with the following PCR primers: Forward (5'-3') TATCACTCTCCTACTTACAG and Reverse (5'-3') ACCTAGAAGGTTGCCTGGCT using touchdown PCR [46] and High-Fidelity Platinum *Taq *polymerase following the manufacturer's protocol (Invitrogen, Carlsbad, CA). Cycle sequencing reactions were carried out using Big Dye Ready Reactions Kits and protocols specified by the manufacturer (Applied Biosystems, Foster City, CA) with the following sequencing primers: Forward (5'-3') TTTCCAAGGACAAATCAGAGA and Reverse (5'-3') GATAGCATTGCGAGACGCTG. These reactions produced complete upstream and downstream sequences of the HVRI that were assembled and aligned in Sequencher 4.8 (GeneCodes Corporation, Ann Arbor, MI). These mtDNA HVRI sequences were deposited in DDBJ/EMBL/GenBank International Nucleotide Sequence Database (accession numbers GU136804-GU136849).

### mtDNA haplotype analysis

Haplotype networks for HVRI mtDNA sequences were generated via the median-joining algorithm of Network 4.5 http://www.fluxus-engineering.com. Because it allows for reticulation, the median-joining approach to the inference of haplotype relationships is appropriate for the analyses of mtDNA control region sequences, which exhibits high levels of homoplasy in humans [47,48]. Hypermutable sites were identified by post-processing using the Steiner maximum parsimony algorithm within Network 4.5 and were excluded from the network analyses.

### Assignment tests

The geographic origins of the LWC chimpanzees were ascertained by smoothed and continuous assignment techniques implemented in SCAT, version 1.0.2 [11]. SCAT implements a Bayesian approach to estimating allele frequencies and assigning a geographic origin to STRP genotype profiles from organisms of unknown origin. These assignments were made by creating a spatial gradient of allele frequencies from georeferenced genotype profiles followed by estimating the likelihood that samples of unknown origin share ancestry with the georeferenced genotype profiles from specific sampling locations within the study area (smoothed assignment method, SAM) and/or originate someplace within the study area independent of where sampling locations are within the study area (continuous assignment method, CAM). The parameters α and β control how correlations between allele frequencies decay with distance. These parameters may be fixed priors or allowed to vary with a thinning parameter that is large enough to return consistent results across independent runs. We completed several initial runs with different combinations of burn-in, iterations and thinning parameters, and found that results between runs were consistent with a thinning parameter of 500 for both the SAM and the CAM analyses. SAM tests were performed with a thinning parameter of 500 with an initial burn-in period of 1,000 replicates and 2,000 iterations for each genotype profile using a leave-one-out cross-validation procedure. Each SAM estimated origin is the product of least five independent runs that were started with different random seeds. The most probable location for each sample was ascertained by the highest mean log-likelihood ratio of each sample's assignment across these independent runs.

CAM tests were also performed to obtain allele frequency estimates from georeferenced samples that were then used to assign samples of unknown origin. The CAM test differs from the SAM test in that the CAM allows each sample's origin to be located anywhere with the study area. We completed CAM tests by specifying a polygon of the study area that included all ten sampling locations of the georeferenced genotype profiles included in this study, as well as all regions of known chimpanzee habitat across Nigeria and Cameroon. The coordinates for this polygon were: N 9.01 E 2.79, N 7.80 E 15.50, N 6.03 E 14.54, N 3.73 E 15.32, N 1.71 E 16.16, N 2.26 E 9.83, N 3.96 E 9.17, N 4.42 E 5.84, N 6.22 E 4.88, N 6.47 E 2.78, N 9.01 E 2.79. CAM tests were performed with a thinning parameter of 500 with an initial burn-in period of 1,000 replicates and 2,000 iterations for each genotype profile using a leave-one-out cross-validation procedure. Each CAM result is the product of least five independent runs that started with a different random seed. For each sample, we obtained CAM results from 10,000 point coordinate estimates. A single point estimate for each sample was determined by taking the median of the coordinates from independent CAM runs. These coordinates were plotted onto a map of the study area in ArcMap 9.2 (ESRI, 1999-2006). We constructed confidence intervals for the CAM estimates for each LWC chimpanzee by plotting 100 coordinates weighted according to their posterior probability that were drawn randomly from across all CAM runs. Finally, we assessed the reliability of CAM estimated origins for LWC chimpanzees inferred to be from southern Cameroon by creating a larger boundary file that included the entire range of *P. t. troglodytes *across central Africa, but not *P. t. ellioti*. The coordinates for this polygon were: N 3.76 E 9.61, N 4.29 E 11.29, N 4.48 E 13.63, N 4.14 E 16.30, N 3.98 E 18.59, N 1.66 E 18.10, N -0.57 E 17.73, N -2.16 E 16.32, N -4.33 E 15.26, N -5.96 E 12.43, N -4.48 E 11.85, N -3.45 E 10.57, N -1.87 E 9.33, N -0.79 E 8.92, N 0.35 E 9.48, N 2.21 E 9.91, N 3.13 E 9.99 and N 3.76 E 9.61. Run parameters for this CAM test included a thinning parameter of 500, a burn-in of 1,000 iterations and 2,000 replicates after the burn-in period.

## Abbreviations

LWC: Limbe Wildlife Centre; HVRI: hypervariable region one of mtDNA; SCAT: smoothed and continuous assignment test; SAM: smoothed assignment test; CAM: continuous assignment test

## Authors' contributions

LG carried out STRP genotyping, participated in data analysis and prepared the manuscript. FL, JAK and AEA collected whole blood samples of the LWC chimpanzees and assisted in data interpretation. SV assisted in data interpretation and manuscript editing. RF, ELG and PDJ participated in project development, project implementation and manuscript preparation. ELG also contributed significantly to data interpretation during manuscript preparation. MKG conceived the project, oversaw project implementation and prepared the manuscript. MKG also carried out data analysis and mtDNA resequencing reactions. All authors read and approved this manuscript.

## Supplementary Material

Additional file 1 Table containing genotype profiles of all samples included in this study in a format appropriate for analysis in SCAT [49]. Click here for file
